# Obesity is a significant risk factor for breast cancer in Arab women

**DOI:** 10.1186/1471-2407-14-788

**Published:** 2014-10-29

**Authors:** Naser Elkum, Taher Al-Tweigeri, Dahish Ajarim, Ali Al-Zahrani, Suad M Bin Amer, Abdelilah Aboussekhra

**Affiliations:** Division of Clinical Epidemiology, Sidra Medical and Research Centre, Doha, Qatar; Department of Medical Oncology, KFSH&RC, Oncology Centre, MBC#64, KSA Riyadh, Saudi Arabia; Department of Molecular Oncology, KFSH&RC, MBC # 03, KSA, Riyadh, Saudi Arabia; Department of Biostatistics, Epidemiology, and Scientific Computing, KFSH&RC, MBC#03, KSA, Riyadh, Saudi Arabia

**Keywords:** Breast cancer, Obesity, Risk factors, Epidemiology, Arab women

## Abstract

**Background:**

Breast cancer (BC) is the most common malignancy and the leading cause of cancer-related death amongst women worldwide. The risk factors of this disease are numerous, and their prevalence varies between racial and ethnic groups as well as geographical regions. Therefore, we sought to delineate the association of socio-demographic, reproductive and life-style related risk factors with breast cancer in the Arab population.

**Methods:**

Unmatched case-control study was conducted in the kingdom of Saudi Arabia using 534 cases of histologically confirmed breast cancer and 638 controls. Controls were randomly selected from primary health care visits and were free of breast cancer. Unconditional logistic regression analysis was performed to estimate odds ratios (ORs) and to examine the predictive effect of each factor on risk for BC. All study participants were interviewed by trained interviewers at hospital (cases) or at primary health care centers (controls).

**Results:**

A total of 1172 women were eligible for this study, of which 281 (24.0%) were aged ≤35 years, 22.9% illiterate, 43.6% employed, 89.5% married, and 38.1% were obese. Grade III tumors constituted 38.4% of cases. Tumor stage I was 7.5%; II, 50.7%; II, 30.9%; IV, 11.1%. We have shown strong association between breast cancer among Arab females and obesity (OR =2.29, 95% CI 1.68-3.13), positive family history of breast cancer (OR =2.31, 95% CI 1.60 – 3.32), the use of hormonal replacement therapy (OR =2.25, 95% CI 1.65 – 3.08), post-menopause (OR =1.72, 95% CI 1.25 – 2.38), lack of education (OR =9.09, 95% CI 5.88 – 14.29), and never breastfeed (OR =1.89, 95% CI 1.19 – 2.94).

**Conclusion:**

These results indicate the presence of classical risk factors established in the western countries, and also some specific ones, which may result from genetic and/or environmental factors. Thereby, these findings will be of great value to establish adequate evidence-based awareness and preventative measures in the Arab world.

## Background

Breast cancer is the most common malignancy and the leading cause of cancer-related death amongst women worldwide [1, 2]. Similarly, in the kingdom of Saudi Arabia (KSA), breast cancer is currently the most common malignancy among females [3–5]. It represents 23% of the total number of cancer cases in the kingdom. The incidence of this disease is witnessing a gradual increase with total cancer cases diagnosed at an average annual age standardized rate (ASR) of 15.6/100,000 [6]. Breast cancer among Saudis is characterized by high aggressiveness, poor clinicopathologic features and early onset [7–9]. Indeed, breast cancer cases tend to be found in younger women with median age of 47 years as compared to 63 in industrialized nations, and with advanced stage of the disease [3, 9, 10]. Young age at onset of breast cancer correlates with a worse prognosis irrespective of the menopausal status, since age remains a risk factor among premenopausal women [11].

A number of breast cancer-related etiological factors have been identified [12–15]. These include genetic, reproductive, environmental and socioeconomic risk factors [16]. In addition, it is becoming increasingly evident that obesity, young age at menarche, late age at first child, short period of lactation and being physically inactive are important risk factors for developing breast cancer in different countries. Furthermore, geographical, racial and ethnic distributions also have major effects on the incidence and the pathophysiology of the disease [1, 17–21]. Notably, studies in developed countries with high prevalence of established risk factors showed that approximately 50% of breast cancer risk is attributable to the established factors [22]. However, the vast majority of these factors were identified and their effects were studied only on western populations. Furthermore, the Gail model on breast cancer risk assessment has been developed in order to predict the number of cancers likely to develop within cohorts of white American women with specific risk factors [23–25]. Therefore, in order to design meaningful prevention strategies, it is very important to identify these factors for each population and geographical location, and to understand the reasons of the observed differences. At present, there is no data available on the breast cancer risk factors for the Arab population. Therefore, in an attempt to identify and better define these risk factors for breast cancer among Arab women, we initiated the present case-control study.

## Methods

### Study population

The study cases were female patients with histological-confirmed primary breast cancer. We started interviewing patients, in the Oncology Department at King Faisal Specialist Hospital & Research Center (KFSH&RC) Riyadh. The controls were Saudi women aged 18 years or older, who visited the primary health care and were cancer free. Volunteers were enrolled in the study during the same calendar period as cases, from all Saudi provinces. Controls were randomly selected and approached while waiting for their doctor’s appointment. Nearly 96% of women approached for the study chose to participate. KFSH&RC is a tertiary care facility and serves as the main referring center for the whole Kingdom of Saudi Arabia (KSA). Therefore, it is conceivable that the cancer pattern seen at KFSH&RC is a reflection to that seen in the whole country. This survey was carried out between June 2007 and August 2012. The study conformed to the principles outlined in the Declaration of Helsinki and was approved by the Research Ethics committee (Office of Research Affairs) at King Faisal Specialist Hospital & Research Center, RAC-2031091.

### Data collection

All study participants were interviewed by trained interviewers at hospital (cases) or at primary health care centers (controls). A structured questionnaire was used to elicit detailed information on demographic factors, menstrual and reproductive history, hormone use, dietary habits, prior disease history, physical activity, tobacco and alcohol use, and family history of cancer. Information on menstrual and reproductive history included age at menarche, menopausal status, age at menopause, pregnancy, and duration of breastfeeding for each live birth. Body height and weight were measured in light indoor clothing without shoes.

Obesity was assessed using BMI cutoffs standard criteria; BMI between 18.5 and 24.9 was considered normal, 25 to 29.9, overweight, and equal to or higher than 30, was considered obese. The education level was stratified into three categories: illiterate, primary or high school education and university studies.

### Data analysis

Frequencies of categorical variables for cases and controls were computed. Tumor characteristics were cross-tabulated between pre-menopause and post-menopause and differences were assessed using χ2 test. Unconditional logistic regression analysis was performed to estimate odds ratios (ORs) and to examine the predictive effect of each factor on risk for breast cancer. Multiple logistic regressions were fitted to adjust for age (≤35 years vs. >35 years), BMI (lean, overweight, obese), marital status (single, ever married), menopause status (pre-menopause, post-menopause), HRT use (yes/no), age at menarche (<13 years vs. ≥13 years), breastfeeding (yes/no), and education levels (illiterate, primary/high school, higher education). Median age at menarche and median age at menopause were chosen as cutoffs values for categorical. For ordered categorical variables, P-value for linear trend was reported. All statistical assessments were two-sided and considered significant with p-value <0.05. Data analysis was carried out using SAS^©^ software (version 9.4; SAS Institute, Cary, NC).

## Results

### Histological features of breast cancer cases in KSA

**Figure 1:**
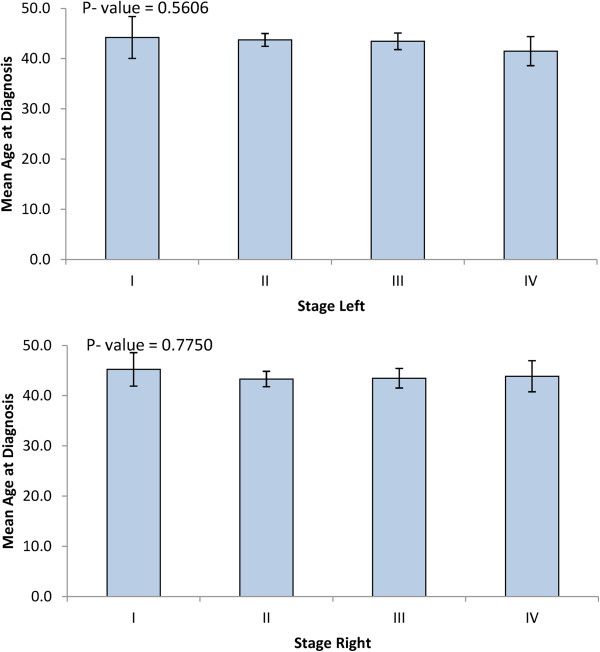
**Least square means of age at diagnosis of breast cancer by stage.**

In the present study we made use of 534 cases of histologically confirmed breast cancer and 638 controls. The age at diagnosis of the breast cancer cases ranged from 22 to 75 years with a mean of 43.6 (SD =8.3) years. While 49.7% of cases were premenopausal, 50.3% of cases were postmenopausal (Table 1). Tumors were of different stages and grades. Tumor stage I was 7.5%; II, 50.7%; II, 30.9%; IV, 11.1%. Figure 1 presents the distribution of age at diagnosis of breast cancer patients according to different classes of tumor stage left/right. The results show early mean age of diagnosis with advanced stage. Grade II and III tumors represented 56.7% and 38.4% of cases, respectively (Table 1). Furthermore, while Her-2 was negative in 54.6% of cases, ER-negative and PR-negative tumors represented 32.4% and 41.2% of cases respectively (Table 1). Interestingly, a significant association was observed between ER status and the menopausal status. Indeed, while 19.6% of premenopausal patients had ER-negative tumors, only 12.9% of postmenopausal cases had ER-negative tumors (*p* =0.0037) (Table 1).

**Table 1:** **Tumor characteristics of the study cases**

Parameter	Total (%) N = 534	Pre-menopause n =266	Post-menopause n =267	***p*** -value
Laterality				0.6945
Left	275 (51.5)	133 (24.9)	142 (26.7)	
Right	250 (46.8)	129 (24.1)	120 (22.6)	
Bilateral	9 (1.7)	4 (0.75)	5 (0.94)	
Stage Left				0.9483
I	14(5.0)	7 (2.5)	7 (2.5)	
II	147 (52.5)	73 (26.1)	74 (26.5)	
III	90 (32.1)	42 (15.0)	48 (17.2)	
IV	29 (10.4)	13 (4.7)	16 (5.7)	
Stage Right				0.0945
I	26 (10.2)	15 (5.9)	11 (4.4)	
II	123 (48.2)	67 (26.4)	56 (22.1)	
III	76 (29.8)	39 (15.4)	36 (14.2)	
IV	30 (11.8)	9 (3.6)	21 (8.3)	
Grade				0.5207
I	25 (4.9)	15 (3.0)	10 (2.0)	
II	285 (56.7)	141 (28.2)	143 (28.6)	
III	193 (38.4)	91 (18.2)	100 (20.0)	
ER				
Negative	173 (32.4)	104 (19.6)	69 (12.9)	
Positive	312 (58.4)	139 (26.2)	170 (32.0)	0.0037
Unknown	49 (9.2)	21 (3.9)	28 (5.3)	
PR				
Negative	220 (41.2)	120 (22.6)	98 (18.5)	
Positive	265 (49.6)	123 (23.2)	141 (26.6)	0.1091
Unknown	49 (9.2)	21 (3.9)	28 (5.3)	
Her_2				
Negative	162 (54.6)	86 (29.3)	74 (25.2)	
Positive	132 (44.4)	77 (26.2)	54 (18.4)	0.5072
Unknown	3 (1.0)	1 (0.34)	2 (0.68)	

Missing a pre-menopausal patient.

### Breast cancer sociodemographic risk factors

A total of 1172 women were eligible for this study, of which 281 (24.0%) were aged ≤35 years, 22.9% illiterate, 43.6% employed, 89.5% married, and 38.1% were obese. Family history of breast cancer, the marital status, education and occupation of breast cancer patients as well as healthy controls were investigated as sociodemographic risk factors of breast cancer among Arabs in KSA. Interestingly, higher proportion of cases (21.9%) than controls (11.4%) reported positive family history of breast cancer with high significance (OR =2.18, 95% CI 1.58 – 2.99). However, there was no significant difference between cases and controls regarding family history of other cancer (*p* =0.9653) (Table 2).

**Table 2:** **Socio-demographical characteristics of the Saudi breast cancer cases and controls**

Socio-demographic characteristics	Cases number (%)	Control number (%)	OR 95% confidence intervals	***P*** -value
Age, years				< 0.0001
≤ 35	80 (15.0)	201 (31.5)	1	
> 35	454 (85.0)	437 (68.5)	2.61 (1.95 – 3.49)	
Family History of Breast Cancer	117 (21.9)	72 (11.4)	2.18 (1.58 – 2.99)	< 0.0001
Family History of Cancer	221 (41.4)	262 (41.3)	1.01 (0.80 – 1.27)	0.9653
Education Level				< 0.0001
Illiterate-no schooling	183 (36.7)	76 (12.1)	1	
1 – 12 years	254 (50.9)	264 (41.9)	0.40 (0.29 – 0.55)	
> 12 years	62 (12.4)	290 (46.0)	0.09 (0.06 – 0.13)	
Ever Married (no)	23 (4.3)	100 (15.7)	0.24 (0.15 – 0.39)	< 0.0001
Never Worked	368 (74.2)	263 (42.2)	3.94 (3.05 – 5.09)	< 0.0001
Ever Smoke (yes)	158 (29.8)	181 (28.8)	1.05 (0.82 – 1.36)	0.6995
BMI, kg/m^2^				
All				< 0.0001
Lean	129 (24.2)	247 (38.7)	1	
Overweight	157 (29.4)	193 (30.3)	1.56 (1.15 – 2.10)	
Obese	248 (46.4)	198 (31.0)	2.40 (1.81 – 3.18)	
Pre-Menopause				< 0.0001
Lean	67 (25.4)	201 (41.5)	1	
Overweight	86 (32.6)	148 (30.6)	1.74 (1.19 – 2.56)	
Obese	111 (42.1)	135 (27.9)	2.47 (1.70 – 3.58)	
Post-Menopause				0.1191
Lean	60 (22.5)	45 (30.2)	1	
Overweight	70 (26.2)	42 (28.2)	1.25 (0.73 – 2.15)	
Obese	137 (51.3)	62 (41.6)	1.66 (1.02 – 2.70)	

*Abbreviations:* *OR* odds ratio, *BMI* body mass index.

Lean: BMI (18.5 - 24.9); Overweight: BMI (25 - 29.9); Obese: BMI (≥30).

**Figure 2:**
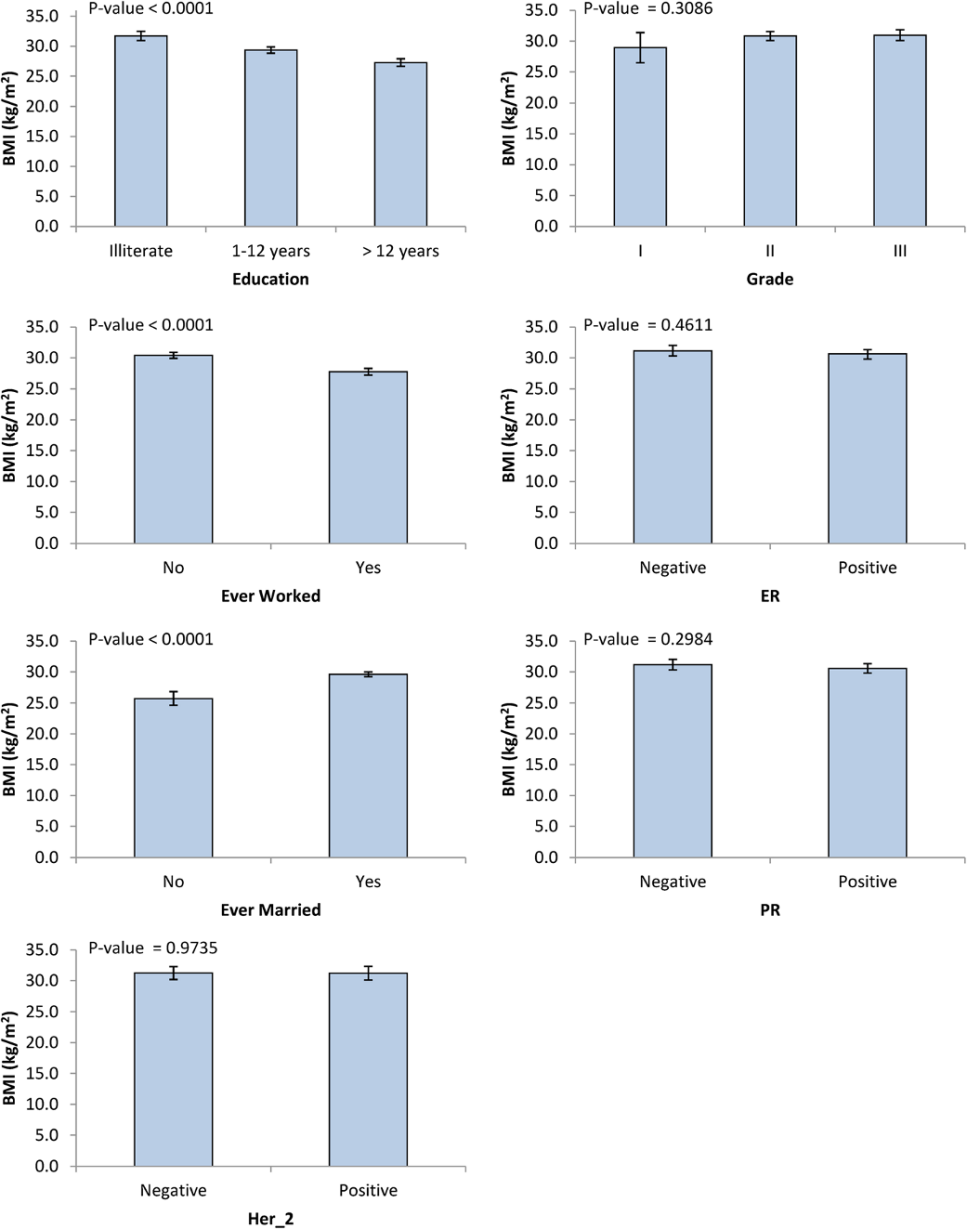
**Least square means of BMI according to various breast cancer risk factors.**

**Figure 3:**
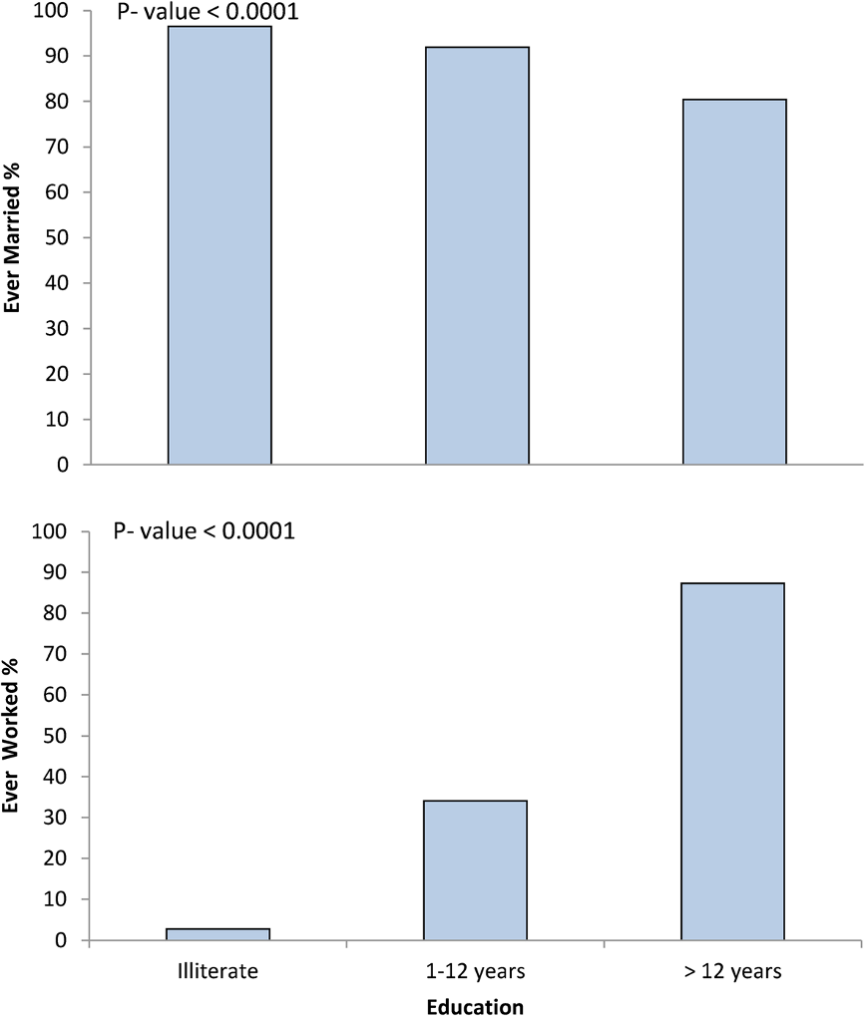
**The proportion of married and employed women by education levels.**

Figure 2 shows significant associations between BMI and each of education, employment status and marital status. Illiterate, unemployed and married women had significantly higher mean BMI (P < 0.0001). Education levels showed high association with marital status and employment in our population (P < 0.0001). Among illiterate women, only 2.8% were working and 96.5% were married; whereas among highly educated women, 87.3% were employed and 80.4% were married (Figure 3).

Furthermore, Table 2 shows significant difference in education and occupation between cases and controls. Indeed, while higher proportion (50.9%) of breast cancer females had received primary education as compared to controls (41.9%), only 12.4% of cases had completed post-high school education as compared to controls 46.0%. This indicates that lower level of education was associated with a significant increased risk of breast cancer in the Arab population (*p* <0.0001). In line with this, 74.2% of the enrolled patients never worked as compared with control subjects (57.8%), indicating that unemployment is significantly related to breast cancer development (OR =3.94, 95% CI 3.05 – 5.09) (Table 2). Moreover, significant difference was found between cases and controls regarding marital status (*p* <0.0001), with the marriage representing a risk factor in this population (Table 2).

### Breast cancer life style risk factors

Alcohol is not available in KSA due to religion issues and therefore its consumption is not a risk factor for this population. Likewise, cigarette smoking was not a common practice among Arab females. Indeed, only 29.8% of cases and 28.8% of controls ever smoked. However, this difference was not statistically significant (*p* =0.6995) (Table 2), suggesting that cigarette smoking was not a risk factor for breast cancer in this population.

### Obesity is a significant breast cancer risk factor in the Arab population

It became clear that obesity plays a major role in development and spread of breast cancer [26, 27]. To elucidate the impact of this important risk factor on the Arab population, we investigated the link between breast cancer and obesity among cases and controls. Table 2 shows clear difference between patients and controls according to their BMI. The proportion of overweight/obese (BMI ≥25) females was significantly higher among breast cancer patients (75.8%) than among healthy controls (61.3%) (OR =1.74 and *p* <0.0001). This clearly shows that obesity is a significant risk factor for breast cancer among Arab women.

When the menopausal status of cases was taken into consideration, we have found 74.7% of pre-menopausal patients were either overweight or obese, while only 58.5% of premenopausal controls exhibited BMI ≥30 with (OR =2.47, *p* <0.0001), indicating two-half fold increase in breast cancer risk among obese premenopausal patients (Table 2). In the post-menopausal females, obese women have 66% increase chance of breast cancer compared to lean post-menopause (Table 2).

### Breast cancer reproductive risk factors

Table 3 shows that 49.7% of Arab breast cancer patients were pre-menopausal. However, more patients (50.3%) were post-menopausal as compared to controls (23.5%). This difference was highly significant (*p* <0.0001), showing high breast cancer risk among post-menopausal females. Most of the Arab females (70.0% cases and 92% controls) reached menopause after 45, and the age at menopause was observed to be associated with increased risk. Indeed, the odds of the risk are 80% lower in women who had menopause after 45 years of age (OR =0.20, P =0.0001). Table 3 shows also that as many as 64.1% of Arab breast cancer patients have used hormonal replacement therapy (HRT) as compared to controls (47.7%), indicating that the use of HRT doubled the chances of developing breast cancer (OR =1.96, *p* <0.0001). On the other hand, no significant difference was found between cases and controls regarding breast feeding (*p* =0.8739) as well as age at menarche in univariate analysis (*p* =0.0767) (Table 3).

**Table 3:** **Reproductive characteristics of Saudi breast cancer cases and controls**

Parameter	Cases number (%)	Controls number (%)	OR 95% confidence intervals	P-values
Menopausal Status				< 0.0001
Pre-menopause	264 (49.7)	484 (76.5)	1	
Postmenopausal	267 (50.3)	149 (23.5)	3.29 (2.56 – 4.22)	
Age at menarche (years)				0.0767
< 13	379 (70.9)	422 (66.1)	1	
≥ 13	155 (29.1)	216 (33.9)	0.80 (0.62 – 1.03)	
Breastfeeding				0.8739
No	38 (8.0)	38 (8.0)	1	
Yes	440 (92.1)	457 (92.0)	0.96 (0.60 – 1.54)	
Use of hormonal replacement therapy				< 0.0001
No	191 (35.9)	329 (52.3)	1	
Yes	341 (64.1)	300 (47.7)	1.96 (1.55 – 2.48)	
Age at menopause (years)				0.0001
< 45	67 (30.0)	7 (8.0)	1	
≥ 45	156 (70.0)	81 (92)	0.20 (0.09 – 0.46)	

Median values were used as cutoff point for age at menopause and age at menarche.

*Abbreviation:* *OR* odds ratio.

### Independent breast cancer risk factors among Arabs

Multivariate logistic regression analysis showed that factors that were independently associated with breast cancer are obesity (OR =2.29, 95% CI 1.68-3.13), positive family history of breast cancer (OR =2.31, 95% CI 1.60 – 3.32), HRT use (OR =2.25, 95% CI 1.65 – 3.08), post-menopause (OR =1.72, 95% CI 1.25 – 2.38), late age of menarche (OR =1.30, 95% CI 0.99 – 1.72), never breastfeed (OR =1.89, 95% CI 1.19 – 2.94), and lack of education (OR =9.09, 95% CI 5.88 – 14.29) (Table 4).

**Table 4:** **Factors independently associated with Saudi breast cancer women - multiple logistic regression**

Parameters	All	Pre-menopausal	Post-menopause
	OR (95% CI)		
BMI, kg/m^2^			
Lean, (18.5 – 24.9)	1	1	1
Overweight/Obese (≥25)	2.29 (1.68 – 3.13)	2.73 (1.79 – 4.18)	2.22 (1.32 – 3.72)
Family history of breast cancer			-
No	1	1	
Yes	2.31(1.60 – 3.32)	5.04 (3.09 – 8.21)	
Age at menarche (years)		-	-
< 13	1		
≥ 13	1.30 (0.99 – 1.72)		
Use of HRT			
No	1	1	1
Yes	2.25 (1.65 – 3.08)	1.73 (1.13 – 2.67)	2.45 (1.53 – 3.92)
Menopausal Status		-	-
Pre-menopause	1		
Post-menopause	1.72 (1.25 – 2.38)		
Breastfeeding			
No	1		1
Yes	0.53 (0.34 – 0.84)		0.20 (0.08 – 0.50)
Education Level			
Illiterate	1	1	1
Primary/High School	0.40 (0.28 – 0.58)	0.11 (0.06 – 0.27)	0.73 (0.44 – 1.20)
Higher education	0.11 (0.07 – 0.17)	0.03 (0.01 – 0.07)	0.21 (0.10 – 0.45)

Model adjusted for age (≤35 years vs. >35 years), BMI (lean, overweight/obese), marital status (single, ever-married), menopause status (pre-menopause, post-menopause), HRT use (yes/no), age at menarche (<13 years vs. ≥13 years), breastfeeding (yes/no), and education levels (illiterate, primary/high school, higher education). All variables in the model are categorical.

*Abbreviations:* *OR* odds ratio, *CI* confidence interval, *BMI* body mass index.

## Discussion

Identification of risk factors and women at high risk for developing breast cancer is highly important for preventing the development of the disease. Owing to the paucity of such data among Arab females, we decided to assess here the strength of association between recognized socio-demographic, reproductive and anthropometric risk factors for breast cancer among Arabs in KSA. This is the first case-control epidemiological investigation on breast cancer risk factors in KSA. We have found that many established risk factors are also associated with breast cancer among Arab females, and therefore coincide with results of Western populations in this regard. Among the well-established risk factors of breast cancer, only obesity, positive family history of breast cancer, use of hormonal replacement therapy, education and employment status were significantly associated with higher risks of breast cancer in this population.

In the present study, we have shown that family history of breast cancer is an independent predictor of breast cancer. Women with a positive family history of breast cancer showed about threefold increased risk of breast cancer (OR =2.31, *p* <0.0001). This parallels what has been previously reported in various populations in different geographical regions [28, 29]. This also reflects the role of genetic and epigenetic modifications at important genes such as *BRCA1* and *BRCA2* in the predisposition to the disease [30]. However, no association was observed between the development of the disease and the presence of other types of cancer in the family.

Using BMI as reference, we found 75.8% of the cases had abnormal weight. Obesity was found to be associated with breast cancer. Overweight/Obese women exhibit more than 2-fold increased risk of breast cancer (OR =2.29) compared to women with normal BMI. Our data support the concept that obesity is a strong risk factor for the disease, which is consistent with previous reports on different populations in various regions [26, 31]. In the Arab population, breast cancer risk was significantly higher among females who were overweight or obese both pre- and post-menopausal (OR =2.73 and OR =2.22 respectively; *p <* 0.0001). On the other hand, obesity was shown to play a protective effect against developing breast cancer in pre-menopausal Caucasian females [26], while other studies have shown no association between obesity and breast cancer risk [29]. This discrepancy may have several explanations, including the implication of genetic and/or environmental factors in the obesity-related development of the disease or physical inactivity. Generally, people in the Gulf countries are physically inactive and spend their leisure time in sedentary activities [32]. Therefore, appropriate measures need to be taken by the healthcare planners to prevent weight gain and obesity that will probably be more cost effective than the treatment of breast cancer and related complications. Furthermore, preventive lifestyle interventions should be targeted at lowering overweight in Arab women.

We have also observed positive association between HRT and breast cancer; confirming the fact that use of HRT increases breast cancer risk. Previous studies have concluded that combinations of estrogen-progesterone increase the risk of breast cancer for women who were treated for at least 5-years [33, 34]. Our data show that using HRT doubles the chance of developing the disease among Arab females.

Breast cancer among Arab females is significantly related with the level of education. Indeed, lack of education was an independent risk factor for breast cancer and was 6 times more common among illiterate females as compared to the highly educated ones, and the risk decreases as the level of education increases. Women with higher education might have healthier lifestyle, which could play a key role in preventing the disease.

Our results showed that breastfeeding has a protective effect against breast cancer development. Cases were less likely than controls to have breastfeed (OR =0.51). This finding is consistent with the results of many other studies [29, 35–37]. Further investigations are recommended to understand the underlying mechanisms of the influence of breastfeeding on breast cancer.

It is well established that breast cancer risk increases with early age at menarche [16]. Surprisingly, we observed an inverse association between early age at menarche and breast cancer risk. Similar result has been recently reported in the Chinese population [38]. This suggests that early age at menarche represents a protective factor in these populations. This may be due to genetic and/or environmental factors.

Finally, this study showed for the first time a number of risk factors associated with incidence of breast cancer among Arab women. The strongest associations were family history of breast cancer, obesity, use of HRT, being post-menopause, illiterate, and having never breastfeed.

Our study had limitations commonly seen in this type of studies. While cases were only from one hospital, which is a tertiary care facility that serves as the main referring center for the whole Kingdom of Saudi Arabia, cases were collected from different regions of the country. This may constitute a bias as to the origin of the patients/controls. Furthermore, controls were all recruited from hospitals. Our sample size of 534 cases and 638 controls may seem rather small for such studies. Another limitation is that BMI, which may change with time, was measured only once for both patients and controls.

## Conclusions

In conclusion, among other risk factors, obesity increases the breast cancer risk in pre- and post-menopause Arab women. Given the fact that obesity is common among this population, there is a need for education campaigns publicizing obesity as an important risk factor for breast cancer and encouraging Arab females to exercise and pursue healthy lifestyle.

## Consent

Written informed consent was obtained from the patient for the publication of this report and any accompanying images.
